# Causal Effects of Yogurt Intake on Gut Microbiota: A European Mendelian Randomization Study

**DOI:** 10.1155/ijog/2921181

**Published:** 2026-03-03

**Authors:** Mengqi Yang, Liping Wang, Peng Zhou, Jiazeng Xia

**Affiliations:** ^1^ Department of General Surgery, The Affiliated Wuxi No. 2 People′s Hospital of Nanjing Medical University, Wuxi, China; ^2^ Department of General Surgery, Institute of General Surgical Research, Jiangnan University Medical Center, Wuxi, China

**Keywords:** gut microbiota, Mendelian randomization, yogurt

## Abstract

**Background:**

Yogurt is reported to maintain the balance of gut microbiota and prevent disease, but the causal relationship remains unclear.

**Methods:**

We selected data from UK Biobank and MiBioGen to perform Mendelian randomization analysis. MR Egger, inverse variance weighted, and so forth were employed to assess the causality between yogurt intake, low‐fat and full‐fat yogurt, and 196 taxa of gut microbiota. Parallelly, low‐fat and full‐fat yogurt were integrated to perform multivariable Mendelian randomization. Then, we summarized preliminary results according to microbiotic taxonomy.

**Results:**

Statistics hinted at the implicit associations between yogurt intake and *Haemophilus* (OR = 2.08), *Clostridium sensu stricto_1* (OR = 1.84), *Peptostreptococcaceae* (OR = 1.53), *Betaproteobacteria* (OR = 0.70), *Bilophila* (OR = 0.58), and *Ruminococcaceae UCG-011* (OR = 0.40), along with the associations between low‐fat yogurt and *Eubacterium ruminantium* (OR = 2.48), *Methanobacteriaceae* (OR = 3.06). The findings were causal and consistent, albeit with some false positive rates.

**Conclusions:**

Yogurt intake suggestively increased the abundance of *Haemophilus*, *Clostridium sensu stricto_1*, and *Peptostreptococcaceae* and decreased the abundance of *Ruminococcaceae UCG-011*, *Betaproteobacteria* and *Bilophila*; low‐fat yogurt suggestively increased the abundance of *Eubacterium ruminantium* and *Methanobacteriaceae*.

## 1. Introduction

Yogurt, a fermented dairy product, contains higher quality protein and micronutrients than milk [1]. It provides health benefits approximate to probiotic supplements [2] but is more flavorful and popular. Thus, yogurt has been widely acknowledged as a component of a healthy diet. It is widely recognized that yogurt intake firstly increased *Lactobacillus*, *Bifidobacterium*, and other beneficial short‐chain fatty acid (SCFA)‐producing microbiota [3]. Also, it remodeled the intestinal environment by improving acidity and calcium [1], which promoted positive metabolism of blood glucose and blood lipid. In addition, yogurt brought in group B vitamins, minerals, and peptides [4] to fulfill nutritional need.

Gut microbiota principally includes bacteria, fungi, and archaea, which colonize the gastrointestinal tract. The number and genome of gut microbiota were 10–100 times more than host cells [5], and they interacted multilaterally with the intestine and other organs to maintain host homeostasis. Studies have proved the imbalance of gut microbiota and metabolite could mediate the gut–brain axis [6], gut–adipose axis, gut–liver axis [7], and so forth, causing various disorders in energy metabolism, immune response, and neuropsychiatric function.

Mendelian randomization (MR) is a study method designed to verify causal relationships. It selects relevant genetic variations as instrument variables (IVs) to substitute for exposures and outcomes [8]. It is known that heredity was determined before delivery, so researchers could minimize the effect of confounders and reverse causality in MR, meanwhile obtaining more randomness compared with controlled studies. Particularly for confounding exposures like lifestyle, MR could provide a larger sample size and stronger evidence [9, 10] for a convincing conclusion.

In recent years, plenty of clinical and animal research has revealed some effects and mechanisms of yogurt consumption. However, there were few randomized studies and no MR studies between yogurt and gut microbiota. So, we perform this study to evaluate the causality within, attempting to discover more functions about microbiotic regulation and disease prevention.

## 2. Materials and Methods

### 2.1. Study Design

**Figure 1:**
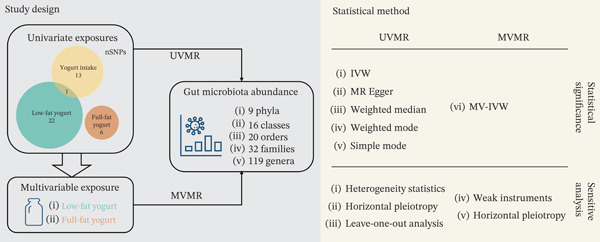
Study design of UVMR and MVMR. We employed yogurt intake, low‐fat and full‐fat yogurt as univariate exposures, low‐fat and full‐fat yogurt as multivariable exposures, and 196 definite taxa of gut microbiota as outcomes. Through statistical tests and sensitive analysis, we assessed the causal association between exposures and outcomes.

This study was conducted in accordance with the STROBE‐MR checklist [11, 12], as Supporting Information 5: Table S1. Based on available database resources, we utilized univariate Mendelian randomization (UVMR) and multivariable Mendelian randomization (MVMR) to assess the causality between yogurt intake and gut microbiota (Figure 1). UVMR took three phenotypes, including yogurt intake and low‐fat and full‐fat yogurt, as respective exposures to estimate their causality with 196 definite taxa of gut microbiota. Subsequently, MVMR was performed to exclude potential confounders like production environment, consumer population, and food additions, further investigating independent effects of nutrient composition (low‐fat/full‐fat) on the outcome. Aforementioned results were clustered according to List of Prokaryotic names with Standing in Nomenclature (LPSN) [13] to conclude causal effects of yogurt intake on taxonomies of gut microbiota.

### 2.2. Data Sources and Instrumental Selection

We accessed three datasets related to yogurt from the Medical Research Council Integrative Epidemiology Unit (MRC‐IEU) consortium [8, 14] and 211 related to gut microbiota from the MiBioGen consortium [15–17] as Table 1. The yogurt intake and low‐fat/full‐fat yogurt diet summaries were from the UK Biobank online 24‐h dietary recall questionnaire. Yogurt intake contained how many pots (half, 1, 2, 3, or more) of yogurt (plain or flavored) were had per day by 97,434 yogurt consumers, as well as their single‐nucleotide polymorphism (SNP) results. Further, the yogurt type (low‐fat or full‐fat) consumed by a large subset of the cohort (89,376 of 97,434) composed the low‐fat/full‐fat yogurt data summaries. Then, MRC‐IEU genome‐wide association study (GWAS) pipeline curated and outputted three exposure GWAS datasets. The results contained 64,949 individuals with European ancestry, including 15,300 cases and 49,649 controls in low‐fat yogurt, as well as 3391 cases and 61,558 controls in full‐fat yogurt. The gut microbiota abundance datasets were obtained from a large‐scale meta‐analysis of human genome–microbiome association conducted by MiBioGen [18]. Through 16 s rRNA sequencing and quantitative microbiome trait loci (mbQTL) mapping, this cohort linked fecal microbiotic abundance to host genome‐wide genotypes. Based on mass published research [7, 19–21], we selected 14,306 European individuals to calculate mbQTL *p* of all 211 gut microbiota taxa as IVs of outcomes. Except for 15 unknown species, 196 taxa were identified relating to host genetic variance. The results included nine phyla, 16 classes, 20 orders, 32 families, and 119 genera.

**Table 1:** Data sources. Three GWAS datasets about yogurt intake, low‐fat, and full‐fat yogurt from UK Biobank were selected as exposures. 211 datasets (including 15 unknown taxon) about gut microbiota abundance from MiBioGen consortium were selected as outcomes.

GWAS ID	Traits	Sample size	Ancestry	Author	Year	Consortium	PMID
Exposure							
ukb‐b‐7753	Yogurt intake	64949	European	Ben Elsworth	2018	MRC‐IEU	NA
ukb‐b‐12936	Type of yogurt eaten: low‐fat yogurt consumers	15300/49649					
ukb‐b‐8659	Type of yogurt eaten: full‐fat yogurt consumers	3391/61558					
Outcome (4/211)							
ebi‐a‐GCST 90016908	Gut microbiota abundance (class Actinobacteria id.419)	14306	European	Kurilshikov A	2021	MiBioGen	33462485
ebi‐a‐GCST 90016909	Gut microbiota abundance (class Alphaproteobacteria id.2379)						
ebi‐a‐GCST 90016910	Gut microbiota abundance (class Bacilli id.1673)						
ebi‐a‐GCST 90017118	Gut microbiota abundance (phylum Verrucomicrobia id.3982)						

Balancing the sample size and statistical power, IVs were selected by following procedures: (1) SNPs which fitted statistical significance threshold (*p* < 10^−5^) were selected as preliminary IVs of yogurt intake. (2) Weak instrument bias of SNPs in UVMR was eliminated through calculating *F* statistic by formula:

F=N–2×R21−R2,

in which *N* represents sample size and

R2=2×beta2×MAF×1−MAF,

representing the proportion of variance in the exposure explained by the genetic variants [22]. Only SNPs with *F* > 10 were retained. (3) SNPs were clumped by MRC‐IEU online linkage disequilibrium (LD) reference panel [8], which contained 1000 European genomes data, to ensure the IVs′ independence to exposures. Applying a threshold of LD *R* ^2^ < 0.001, we retained only the SNP with lowest *p* within 10,000 kb distance. (4) SNPs which could potentially affect outcomes via nonyogurt traits or diseases were excluded. They were confirmed by querying each in PhenoScanner [23, 24] at the threshold (*p* < 5 × 10^−8^). (5) Proxy SNPs were adopted by LD *R* − square > 0.8 when the originals were not available in the outcomes.

### 2.3. Statistical Analysis

A variety of approaches were employed to evaluate the significance and sensitivity of the causality between yogurt intake and gut microbiota. In UVMR, significance *p* was calculated by MR Egger, weighted median, inverse variance weighted (IVW), weighted mode, and simple mode for multiple IVs. Final results were determined by specific method under different circumstances. For instance, we applied IVW when neither heterogeneity nor horizontal pleiotropy existed. Multiplicative random‐effect inverse variance weighted (IVW‐MRE) was employed when only heterogeneity existed. MR Egger was applied when horizontal pleiotropy existed, since its intercept term represented the pleiotropy. Only results with *p* < 0.05 in appropriate method and concordant *β* across multiple methods were retained. As for single IV results, we could only apply Wald ratio method. In MVMR, we utilized IVW MVMR model (MV‐IVW) to estimate the direct effect of each exposure. Finally, all results were subjected to Benjamini–Hochberg (BH) [25] false discovery rate (FDR) correction, adopting an adjusted significance threshold of *p* < 0.05.

Heterogeneity statistics and horizontal pleiotropy were performed in UVMR. We set Cochran′s *Q* statistics threshold (*p* < 0.05) to confirm the heterogeneity between IVs. Additionally, “leave‐one‐out” analysis was taken to detect the heterogeneous IVs. As for horizontal pleiotropy, the intercept term in MR Egger regression aforementioned could be a helpful indicator. Its deviation from zero at the threshold (*p* < 0.05) indicated the presence of pleiotropic bias. About MVMR, we calculated *F* statistic to eliminate weak instrument by *F* > 10, as follows [22]:

F=N–k–1/k×R2/1−R2,

in which k represents the number of IVs and other variates are mentioned before. Besides, a modified form of Cochran′s *Q* statistic was also applied to evaluate horizontal pleiotropy in MVMR. After the aforementioned tests, we employed Steiger directionality test [26] to ensure causal direction (*p* < 0.05) in both UVMR and MVMR.

In the last stage, we summarized above statistical results according to LPSN [13]. Only the most subordinate taxon was kept as representative to unveil yogurt causal effects on specific genera. Then, the superordinate taxa were taken to portray a clustering tree, visualizing yogurt effect in phylogenetic relationship.

## 3. Results

### 3.1. IV Selection

We obtained 13 SNPs for UVMR and 16 for MVMR as Supporting Information 6: Table S2 and Supporting Information 7: Table S3, following the IVs’ selecting procedures. Due to restricted sample size in UVMR, only IVs of yogurt intake associated strongly enough with exposure. The SNP rs149870452 relating to cause of death: dilated cardiomyopathy (OR = 0.91, 95% CI: 0.87–0.94, *p* = 1.3 × 10^−6^) was eliminated. In MVMR, we excluded rs2517506 relating to multiple immunocyte counts [27] and autoimmune diseases [28, 29], rs317656 relating to multiple immunocyte percentages, rs6888979 relating to height and rs9604488 relating to red cell distribution width [27].

### 3.2. UVMR

**Figure 4:**
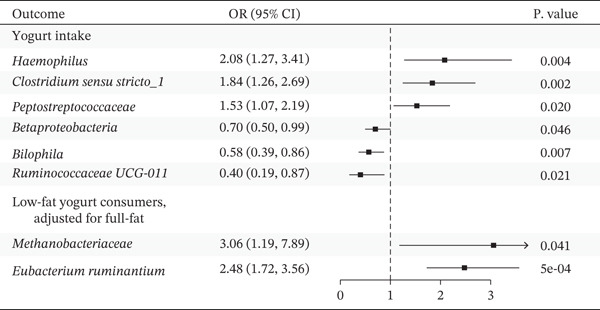
Forest plot of summarized microbiota in UVMR and MVMR. OR, 95% CI, and *p* were calculated by IVW method. As shown, yogurt intake increased the abundance of *Haemophilus*, *Clostridium sensu stricto_1*, and *Peptostreptococcaceae* and decreased the abundance of *Betaproteobacteria*, *Bilophila*, and *Ruminococcaceae UCG-011*. Low‐fat yogurt increased the abundance of *Methanobacteriaceae* and *Eubacterium ruminantium*.

After eliminating biases of heterogeneity (Figure 2, Supporting Information 1: Figure S1, and Supporting Information 8: Table S4), horizontal pleiotropy (Figure 3 and Supporting Information 2: Figure S2), outlier (Supporting Information 3: Figure S3 and Supporting Information 4: Figure S4), weak instrument and causal direction (Supporting Information 8: Table S4), family *Peptococcaceae* with both heterogeneity (Q_pval = 0.025, IVW method) and horizontal pleiotropy (*p* = 0.024) were excluded. There were 12 taxa of gut microbiota causally related to yogurt intake, as Supporting Information 9: Table S5. Then, we summarized one class, one family, and four specific genera by LPSN taxonomy, as shown in Figure 4 and Table 2. In IVW method, the statistics of related microbiota were approximate. There were four typical taxa with significant difference in both IVW and weighted median method. They were genus *Haemophilus* (OR = 2.08, 95% CI: 1.27–3.41, *p* = 3.50 × 10^−3^), representing family *Pasteurellaceae* and order *Pasteurellales*; genus *Clostridium sensu stricto_1* (OR = 1.84, 95% CI: 1.26–2.69, *p* = 1.68 × 10^−3^), representing family *Clostridiaceae_1*; family *Peptostreptococcaceae* (OR = 1.53, 95% CI: 1.07–2.19, *p* = 0.02); and genus *Ruminococcaceae UCG-011* (OR = 0.40, 95% CI: 0.19–0.87, *p* = 0.02). Besides, class *Betaproteobacteria* (OR = 0.70, 95% CI: 0.50–0.99, *p* = 4.61 × 10^−2^) and genus *Bilophila* (OR = 0.58, 95% CI: 0.39–0.86, *p* = 7.41 × 10^−3^) were significant in IVW method, while the latter representing family *Desulfovibrionaceae*, order *Desulfovibrionales*, and class *Deltaproteobacteria*.

Figure 2Leave‐one‐out analysis of summarized microbiota in UVMR. Calculating without each SNP successively, the overall results were consistent and indicated no heterogeneity. (a) Genus *Haemophilus*, (b) genus *Clostridium sensu stricto_1*, (c) family *Peptostreptococcaceae*, (d) class *Betaproteobacteria*, (e) genus *Bilophila*, and (f) genus *Ruminococcaceae UCG-011.*

**Figure:**
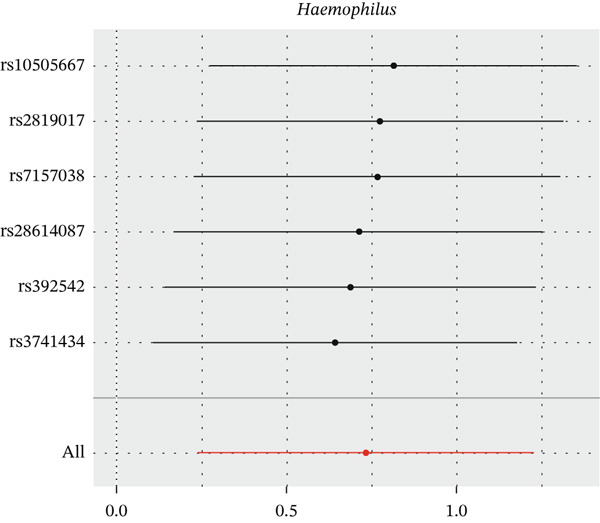
(a)

**Figure:**
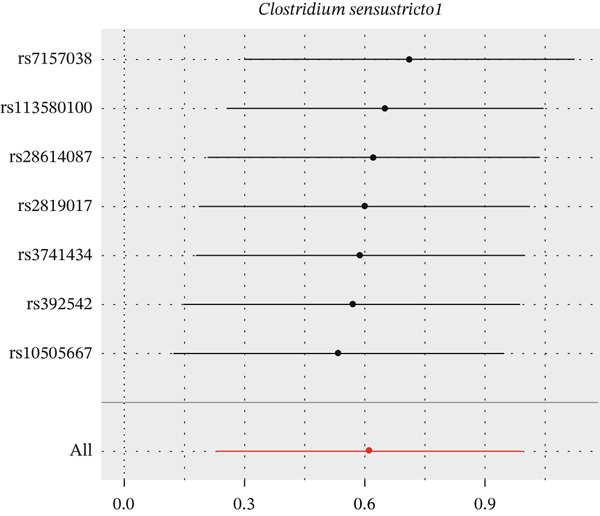
(b)

**Figure:**
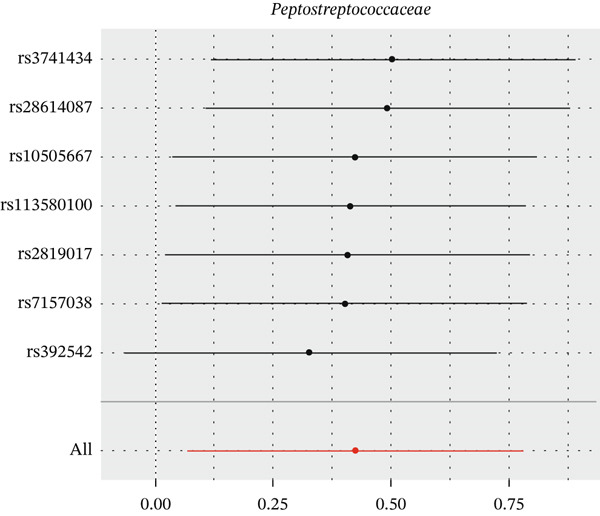
(c)

**Figure:**
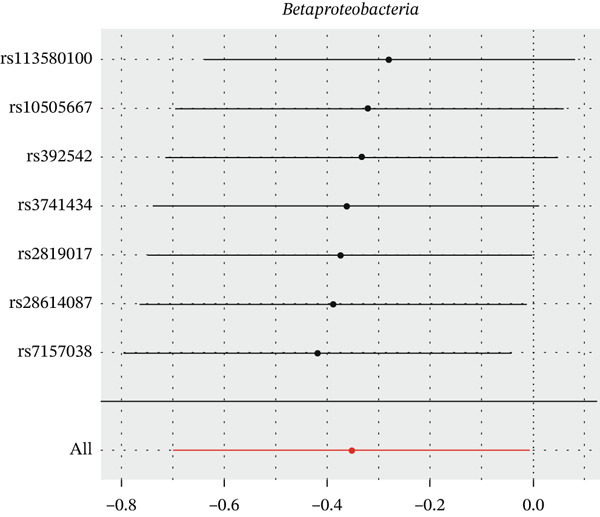
(d)

**Figure:**
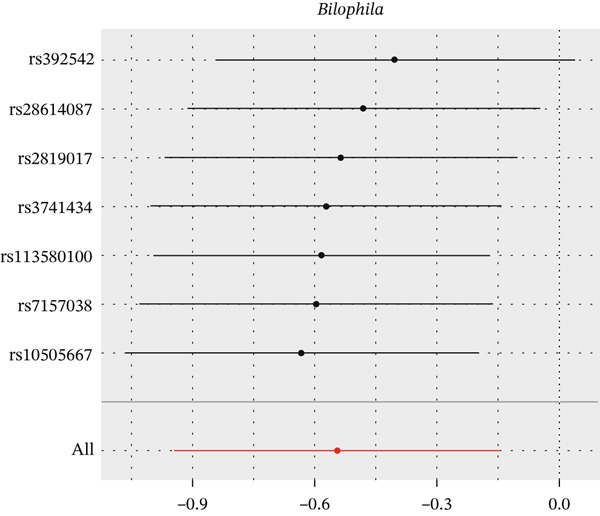
(e)

**Figure:**
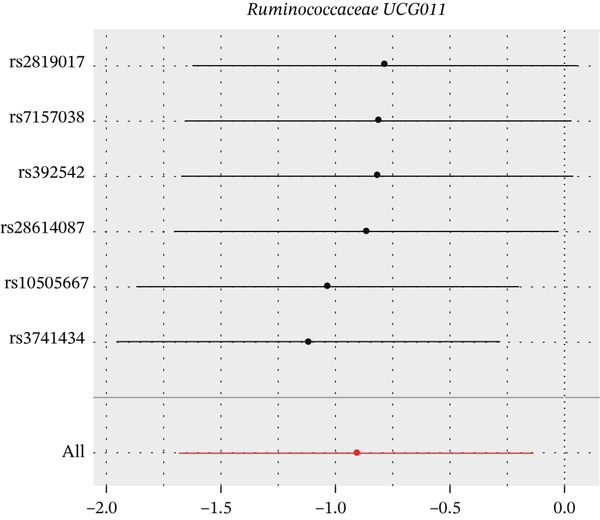
(f)

Figure 3Scatter plot of summarized microbiota in UVMR. The tendencies were consistent in all five regression methods, represented by different color of lines. (a) Genus *Haemophilus*, (b) genus *Clostridium sensu stricto_1*, (c) family *Peptostreptococcaceae*, (d) class *Betaproteobacteria*, (e) genus *Bilophila*, and (f) genus *Ruminococcaceae UCG-011.*

**Figure:**
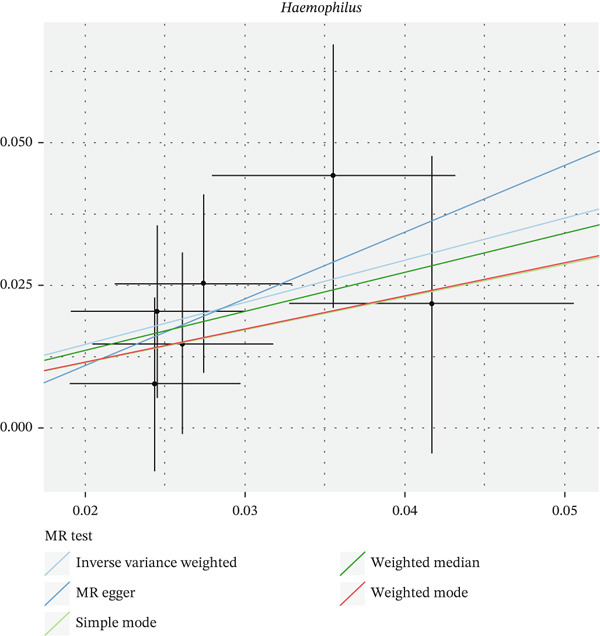
(a)

**Figure:**
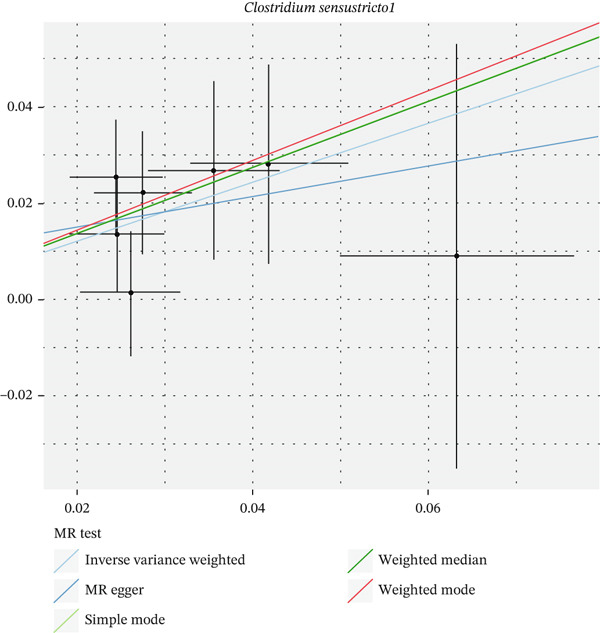
(b)

**Figure:**
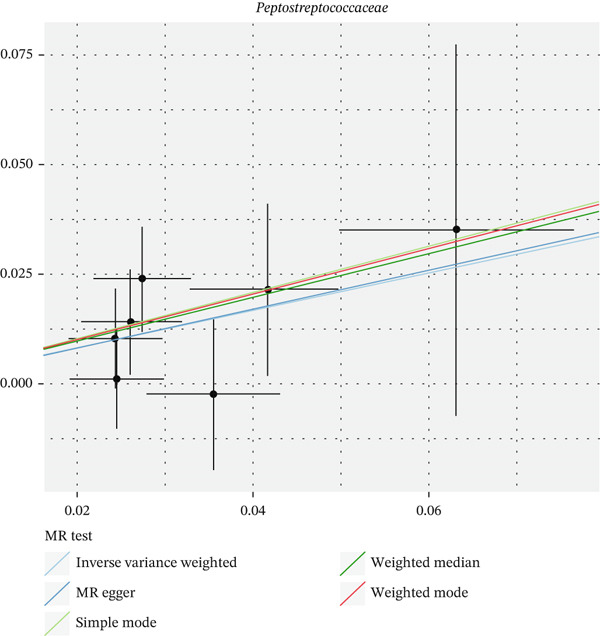
(c)

**Figure:**
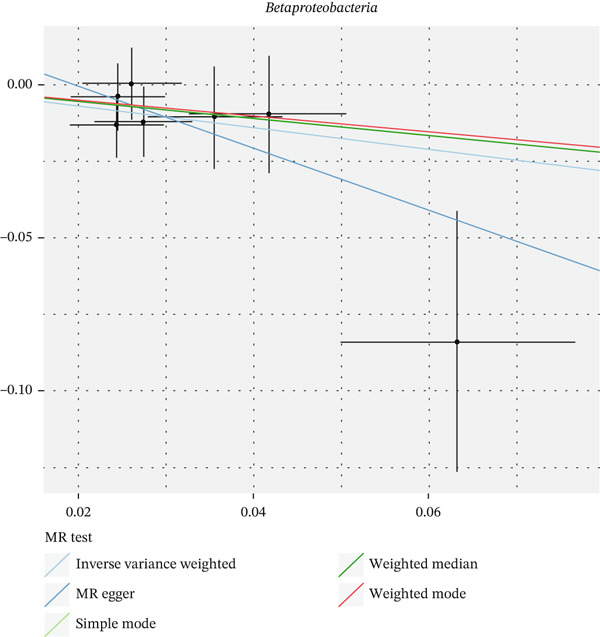
(d)

**Figure:**
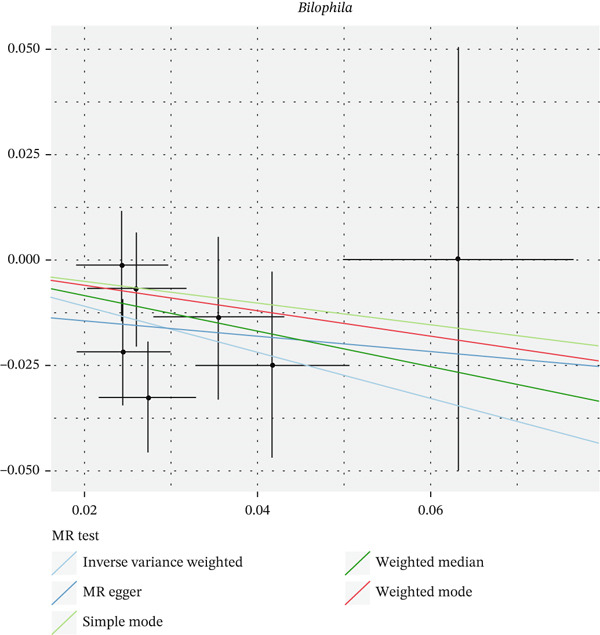
(e)

**Figure:**
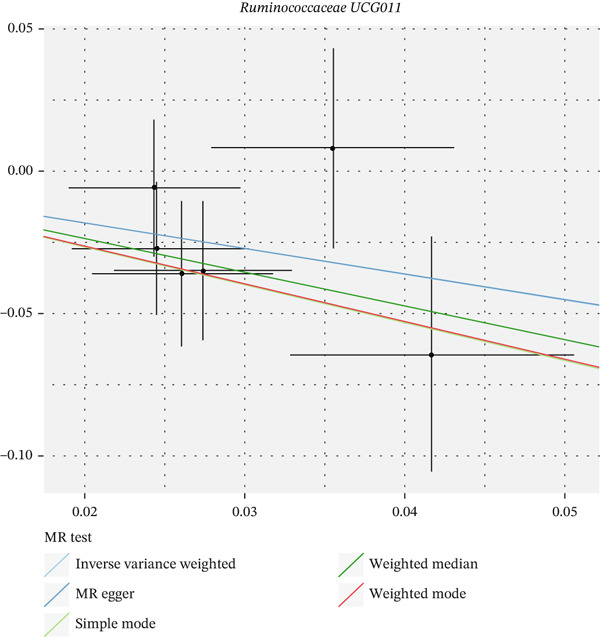
(f)

**Table 2:** Summarized microbiota in UVMR and MVMR. *β*, se, and *p* were calculated by IVW method.

Exposure	Outcome	nSNP	Method	*β*	se	*p*
Yogurt intake	*Haemophilus*	6	IVW	0.73	0.25	3.50 × 10^−3^
	*Clostridium sensu stricto_1*	7		0.61	0.19	1.68 × 10^−3^
	*Peptostreptococcaceae*	7		0.42	0.18	0.02
	*Betaproteobacteria*	7		−0.35	0.18	4.61 × 10^−2^
	*Bilophila*	7		−0.54	0.20	7.41 × 10^−3^
	*Ruminococcaceae UCG-011*	6		−0.91	0.39	0.02

Low‐fat yogurt	*Methanobacteriaceae*	16	MV‐IVW	1.12	0.48	0.04
	*Eubacterium ruminantium*			0.91	0.18	4.76 × 10^−3^

### 3.3. MVMR

All the results relating to full‐fat yogurt were excluded due to the weak instrument strength (*F* < 10, Supporting Information 8: Table S4). We summarized two results in low‐fat yogurt as Figure 4 and Table 2, genus *Eubacterium ruminantium* (OR = 2.48, 95% CI: 1.72–3.56, *p* = 4.76 × 10^−4^) and family *Methanobacteriaceae* (OR = 3.06, 95% CI: 1.19–7.89, *p* = 0.04), while the latter representing order *Methanobacteriales*, class *Methanobacteria*, and phylum *Methanobacteriota*.

### 3.4. Phylogenetic Rree

**Figure 5:**
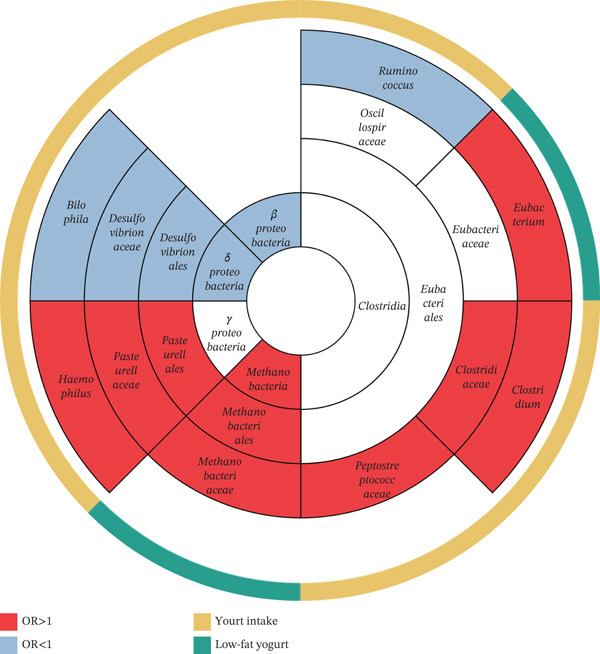
Phylogenetic tree of summarized microbiota. Rings from center to edge successively indicated class, order, family and genus of gut microbiota. Inner colors (red/blue) represented abundance variation (increase/decrease), and outer colors (yellow/green) represented exposure type (yogurt/low‐fat yogurt). Overall, yogurt intake mostly affected abundance of phylum *Pseudomonadota* and order *Eubacteriales*.

According to MR results, we clustered a phylogenetic tree (Figure 5) to reveal yogurt causal effects on taxonomy. The principal alterations occurred in order *Eubacteriales*, whose abundance were mostly increased. Alterations also occurred in three classes of phylum *Pseudomonadota*, but we could not find consistent trends among them.

## 4. Discussion

### 4.1. Limitation

There were still several limitations in our study. Due to limited sample size (64,949), applying standard threshold (*p* < 5 × 10^−8^) would lead to no available IVs. Thus, we deliberately employed a more lenient threshold (*p* < 10^−5^) to obtain sufficient IVs, at the cost of introducing weak instrument bias and less statistical power. As a compensatory measure, *F* statistic of each result was tested. Furthermore, restricted sample size and closely related gut microbiota inevitably reduced the significance of our findings, which led to zero significant result even following lenient BH FDR correction (Supporting Information 8: Table S4). However, we noted that original results were consistent on not only taxonomy as Figure 5, but also published studies mentioned later. Thus, we retained these findings with OR and 95% CI to better assess the magnitude and precision. Additionally, we could not make conclusion in other ethnic populations since the European data source. All those limitations restricted our study to exploratory rather than definitive conclusions. We would continue seeking appropriate data sources and research methods to further study the biomedical value of yogurt.

### 4.2. Perspective

Our MR study demonstrated yogurt intake was causally associated with increasing abundance of genus *Haemophilus*, genus *Clostridium sensu stricto_1*, and family *Peptostreptococcaceae* and decreasing abundance of class *Betaproteobacteria*, genus *Bilophila*, and genus *Ruminococcaceae UCG-011*. Furthermore, we found the causal association between low‐fat yogurt and increasing abundance of genus *Eubacterium ruminantium* and family *Methanobacteriaceae*. These alterations were similar to several studies about probiotic, prebiotic, and synbiotic treatment [2, 30, 31], specifically in modulating *Eubacterium*, *Clostridiales*, *Eubacterium ruminantium*, and *Ruminococcaceae UCG-011*. Relevant studies have revealed that yogurt mediated gut microbiota by its probiotic colonies and metabolites. *Lactobacillus*, *Bifidobacterium*, and other probiotics contained in yogurt could not only colonize into the gut flora [32], but also change the intestinal environment through bacterial metabolism. Simultaneously, yogurt intake increased the abundance of group B vitamins, peptides, and minerals to strengthen the beneficial remodeling for intestinal environment [1, 4, 33].

Recent research has also associated these microbiotic alterations to numerous diseases. They assumed the SCFAs [3] and branched chain hydroxy acids [33] as metabolic mediators to benefit energy metabolism. Decrease in *Haemophilus* correlated to Type 2 diabetes [34] including pregestational diabetes [35], while the increase linked with athlete [3] and high skeletal muscle mass [36] instead. Decrease in *Peptostreptococcaceae* correlated to Type 2 diabetes [37]. Decrease in *Betaproteobacteria* linked with subcutaneous and visceral adipose tissue production [7]. Increase in *BiIophiIa* correlated to Type 2 diabetes [38] and child obesity [39]. Further research indicated that remodeled intestinal environment produced interleukin [40], proinflammatory cytokine [41], and sIgA [31] as immunological mediators, driving downstream TNF, TGF‐*β* [40], and so forth, pathways to regulate inflammation and immunologic disorder. Decrease in *Haemophilus* correlated to inflammatory bowel disease [34]. Decrease in *Clostridium sensu stricto_1* correlated to upper urinary urolithiasis [19]. Decrease in *Peptostreptococcaceae* correlated to primary biliary cholangitis [20], myasthenia gravis [42] and non‐small‐cell lung cancer [43]. Increase in *Ruminococcaceae UCG-011* correlated to age‐related macular degeneration [21]. Decrease in *Methanobacteriaceae* correlated to knee osteoarthritis [44] and irritable bowel syndrome [45]. Decrease in *Eubacterium ruminantium* correlated to appendicitis [46].

The immunological mediators also regulated neuropsychiatric disorders via gut–brain axis potentially. Decrease in *Haemophilus* correlated to Parkinson′s disease [6]. Decrease in *Clostridium sensu stricto_1* correlated to autism spectrum disorder [47]. Decrease in *Peptostreptococcaceae* correlated to attention deficit hyperactivity disorder [48]. Increase in *Betaproteobacteria* correlated to epilepsy [49], bipolar disorder [50], and narcolepsy Type 1 [51]. Increase in *BiIophiIa* correlated to cognition impairment [52] and Parkinson′s disease [6]. Special attention needed to be paid to yogurt intake during infancy. The microbiotic alterations were partly contrary to breastfeeding [53, 54] and positively correlated to atopic dermatitis [55].

## 5. Conclusions

Yogurt intake suggestively increased the abundance of *Haemophilus*, *Clostridium sensu stricto_1*, and *Peptostreptococcaceae*, while decreased the abundance of *Ruminococcaceae UCG-011*, *Betaproteobacteria* and *Bilophila*. Low‐fat yogurt suggestively increased the abundance of *Eubacterium ruminantium* and *Methanobacteriaceae*. The findings were causal and consistent, although they did not survive FDR correction.

## Author Contributions

Jiazeng Xia: conceptualization, funding acquisition, and supervision; Mengqi Yang: software, visualization, and writing—original draft; Liping Wang: resources and methodology; Peng Zhou: writing—review and editing.

## Funding

This work was supported by the Key Project of Scientific Research from Jiangsu Commission of Health, ZDB2020026; Wuxi Taihu Lake Talent Plan, Team in Medical and Health Profession; and Wuxi Medical Key Discipline Construction Project, Medical Development Discipline.

## Ethics Statement

No specific ethical approval was required for this study since the summary‐level GWAS data were publicly accessible. Ethical approval for the original GWASs can be found in the corresponding GWAS publications cited below.

## Conflicts of Interest

The authors declare no conflicts of interest.

## Supporting Information

Additional supporting information can be found online in the Supporting Information section.

## Supporting information


**Supporting Information 1** Figure S1: Leave‐one‐out analysis of preliminary microbiota in UVMR. The results were consistent after excluding SNP successively and indicated no heterogeneity. (A) class *Deltaproteobacteria*, (B) family *Clostridiaceae_1*, (C) family *Desulfovibrionaceae*, (D) family *Pasteurellaceae*, (E) order *Desulfovibrionales*, and (F) order *Pasteurellales.*

**Supporting Information 2** Figure S2: Scatter plot of preliminary microbiota in UVMR. The results were consistent in all regression methods, represented by different colors of lines. (A) class *Deltaproteobacteria*, (B) family *Clostridiaceae_1*, (C) family *Desulfovibrionaceae*, (D) family *Pasteurellaceae*, (E) order *Desulfovibrionales*, and (F) order *Pasteurellales.*

**Supporting Information 3** Figure S3: Forest plots of all microbiota in UVMR. It visualized the effect of each SNP by Wald ratio method and overall result to detect outlier. The results were visually coherent and consistent. (A) class *Betaproteobacteria*, (B) class *Deltaproteobacteria*, (C) family *Clostridiaceae_1*, (D) family *Desulfovibrionaceae*, (E) family *Pasteurellaceae*, (F) family *Peptostreptococcaceae*, (G) genus *Bilophila*, (H) genus *Clostridium sensu stricto_1*, (I) genus *Haemophilus*, (J) genus *Ruminococcaceae UCG-011*, (K) order *Desulfovibrionales*, and (L) order *Pasteurellales.*

**Supporting Information 4** Figure S4: Funnel plots of all microbiota in UVMR. It measured the symmetry of IVs’ *β* and standard error (se) to detect outlier. No outlier was visually detected. (a) class *Betaproteobacteria*, (b) class *Deltaproteobacteria*, (c) family *Clostridiaceae_1*, (d) family *Desulfovibrionaceae*, (e) family *Pasteurellaceae*, (f) family *Peptostreptococcaceae*, (g) genus *Bilophila*, (h) genus *Clostridium sensu stricto_1*, (i) genus *Haemophilus*, (j) genus *Ruminococcaceae UCG-011*, (k) order *Desulfovibrionales*, (l) order *Pasteurellales.*

**Supporting Information 5** Table S1: STROBE‐MR checklist of recommended items to address in reports of Mendelian randomization studies.

**Supporting Information 6** Table S2: SNP information in UVMR and MVMR, including chromosomal location, allele, and eaf.

**Supporting Information 7** Table S3: SNP statistics in UVMR an d MVMR, including *β*, se, *p*, and *F* statistic.

**Supporting Information 8** Table S4: Heterogeneity statistics (IVW and MR Egger), horizontal pleiotropy test, *F* statistic, Steiger directionality test, and *p* adjusted (BH) of UVMR and MVMR.

**Supporting Information 9** Table S5: All causal results of UVMR and MVMR, including *β*, se, and *p* calculated by five methods.

## Data Availability

All GWAS datasets and software in this study are publicly available. The summary results of yogurt intake (GWAS ID: ukb‐b‐7753; UKB ID: 102090), low‐fat (GWAS ID: ukb‐b‐12936; UKB ID: 20106) and full‐fat yogurt (GWAS ID: ukb‐b‐8659; UKB ID: 20106) and gut microbiota abundance (GWAS ID: from ebi‐a‐GCST90016908 to ebi‐a‐GCST90017118) can be downloaded from IEU OpenGWAS project [8, 14]. All analyses in this study were performed by R packages TwoSampleMR (Version 0.5.7) [8] and MVMR (Version 0.4) [56] in R (Version 4.3.1) [57] and RStudio software (Version 6.0.421) [58].
