# Supplementary figures and images for: Causal Effects of Yogurt Intake on Gut Microbiota: A European Mendelian Randomization Study
Source: Int J Genomics. 2026 Mar 3;2026:2921181. doi: 10.1155/ijog/2921181 (PMC12957542; doi:10.1155/ijog/2921181)

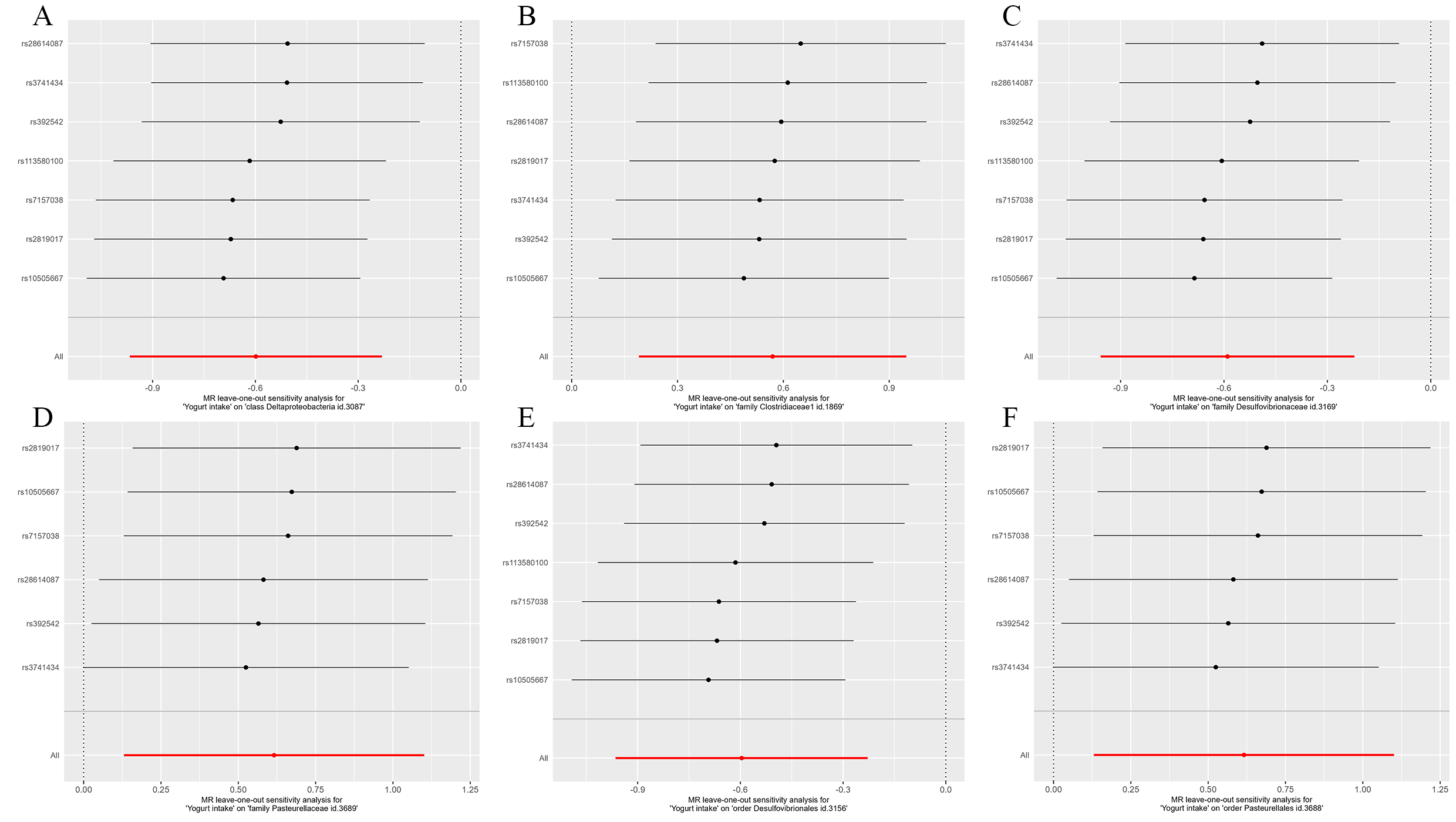

Supplement: Supplementary file 1 — Supporting Information 1 Figure S1: Leave‐one‐out analysis of preliminary microbiota in UVMR. The results were consistent after excluding SNP successively and indicated no heterogeneity. (A) class Deltaproteobacteria, (B) family Clostridiaceae_1, (C) family Desulfovibrionaceae, (D) family Pasteurellaceae, (E) order Desulfovibrionales, and (F) order Pasteurellales. [file IJOG-2026-2921181-s004.tif]

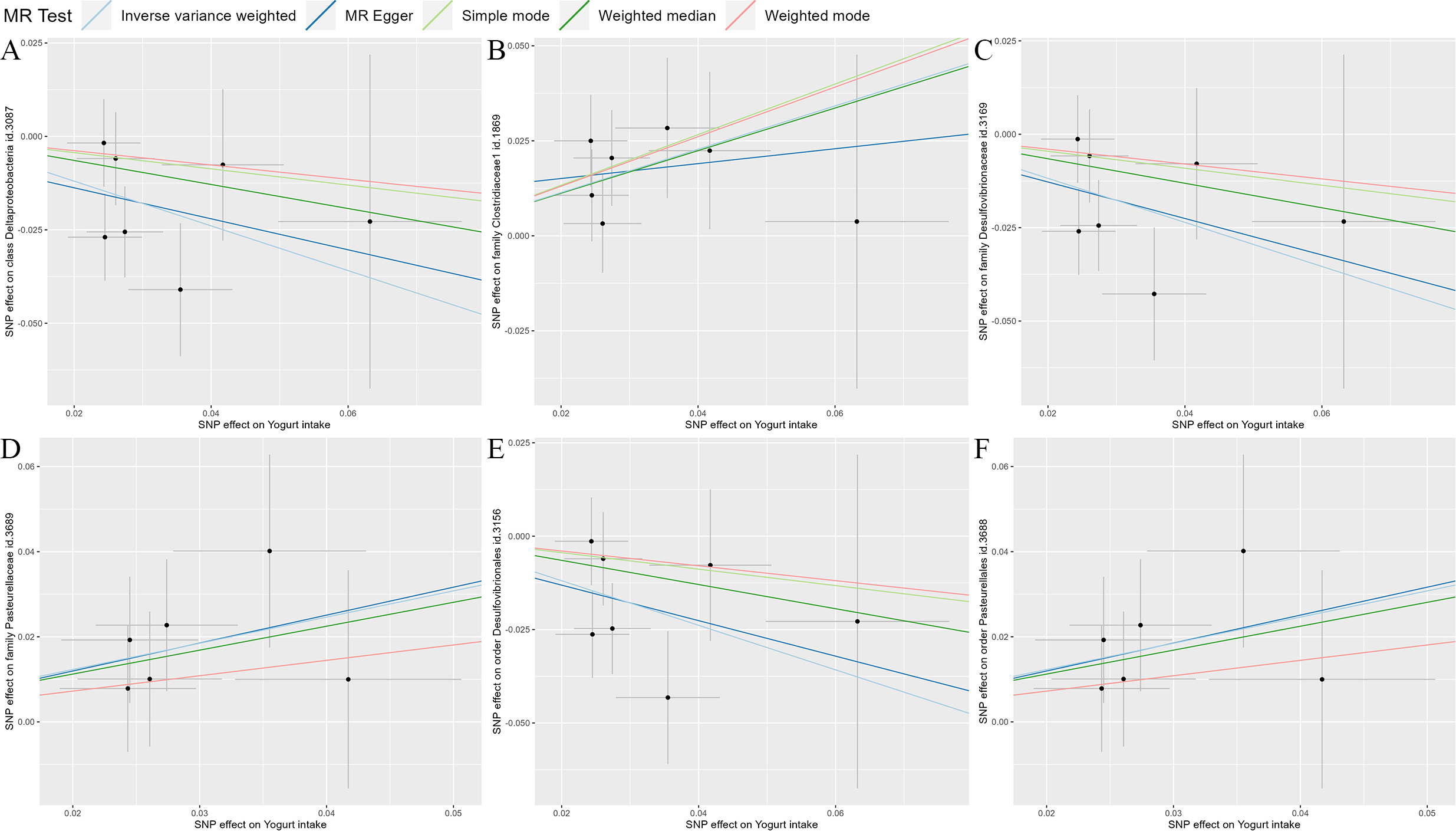

Supplement: Supplementary file 2 — Supporting Information 2 Figure S2: Scatter plot of preliminary microbiota in UVMR. The results were consistent in all regression methods, represented by different colors of lines. (A) class Deltaproteobacteria, (B) family Clostridiaceae_1, (C) family Desulfovibrionaceae, (D) family Pasteurellaceae, (E) order Desulfovibrionales, and (F) order Pasteurellales. [file IJOG-2026-2921181-s005.tif]

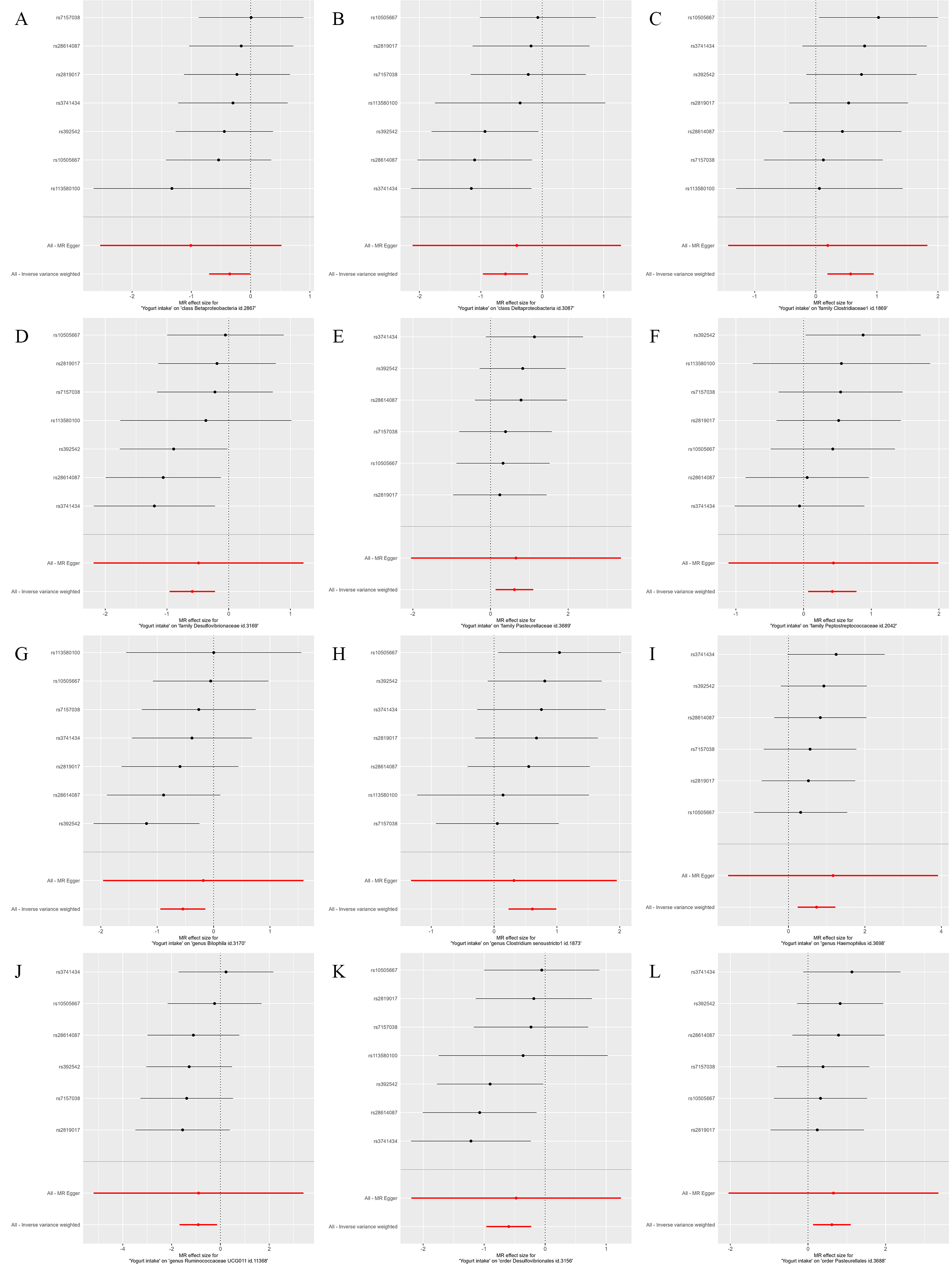

Supplement: Supplementary file 3 — Supporting Information 3 Figure S3: Forest plots of all microbiota in UVMR. It visualized the effect of each SNP by Wald ratio method and overall result to detect outlier. The results were visually coherent and consistent. (A) class Betaproteobacteria, (B) class Deltaproteobacteria, (C) family Clostridiaceae_1, (D) family Desulfovibrionaceae, (E) family Pasteurellaceae, (F) family Peptostreptococcaceae, (G) genus Bilophila, (H) genus Clostridium sensu stricto_1, (I) genus Haemophilus, (J) genus Ruminococcaceae UCG-011, (K) order Desulfovibrionales, and (L) order Pasteurellales. [file IJOG-2026-2921181-s001.tif]

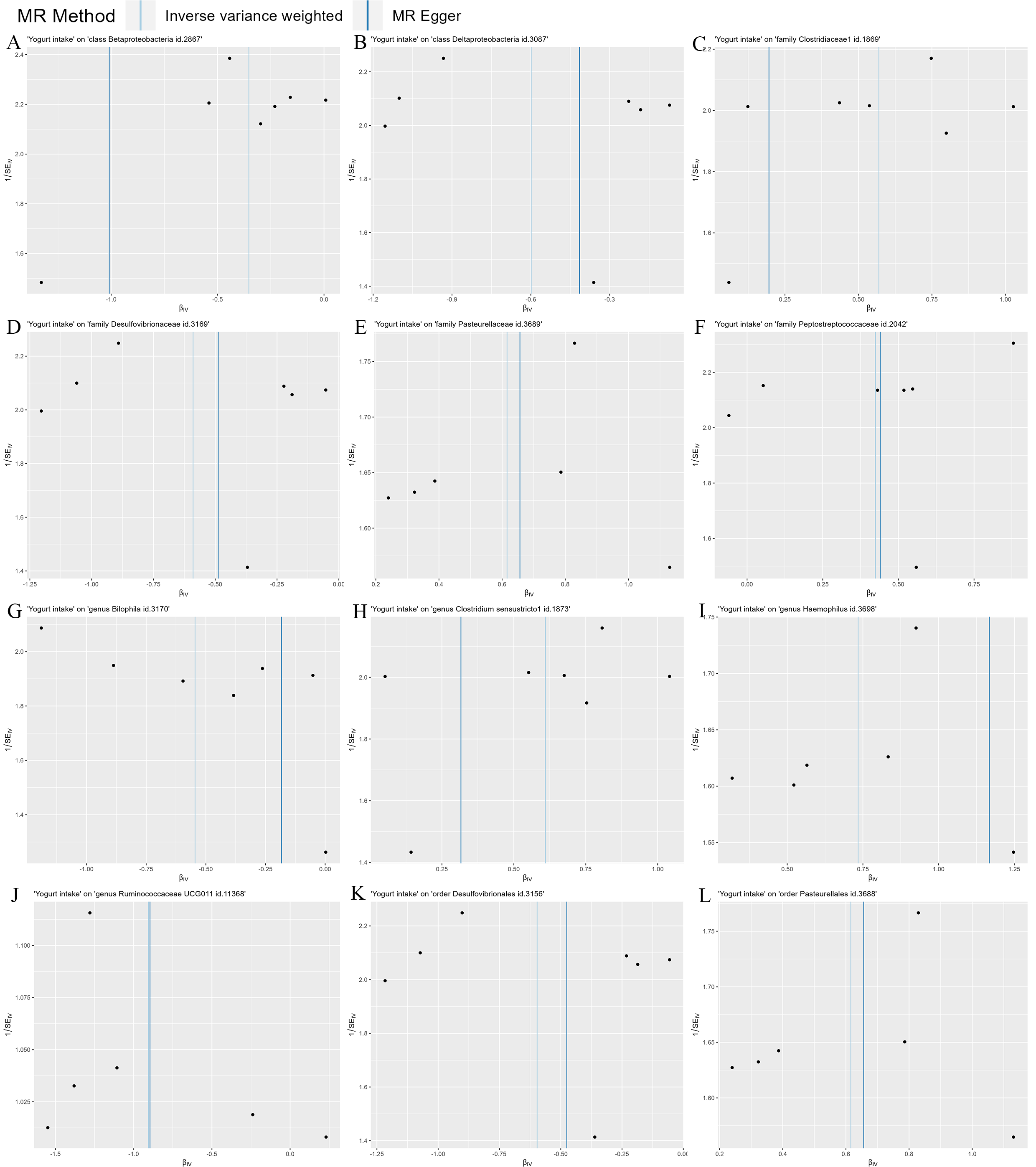

Supplement: Supplementary file 4 — Supporting Information 4 Figure S4: Funnel plots of all microbiota in UVMR. It measured the symmetry of IVs’ β and standard error (se) to detect outlier. No outlier was visually detected. (a) class Betaproteobacteria, (b) class Deltaproteobacteria, (c) family Clostridiaceae_1, (d) family Desulfovibrionaceae, (e) family Pasteurellaceae, (f) family Peptostreptococcaceae, (g) genus Bilophila, (h) genus Clostridium sensu stricto_1, (i) genus Haemophilus, (j) genus Ruminococcaceae UCG-011, (k) order Desulfovibrionales, (l) order Pasteurellales. [file IJOG-2026-2921181-s002.tif]
