# Supplementary material for: Causal Effects of Yogurt Intake on Gut Microbiota: A European Mendelian Randomization Study
Source: Int J Genomics. 2026 Mar 3;2026:2921181. doi: 10.1155/ijog/2921181 (PMC12957542; doi:10.1155/ijog/2921181)
Supplement: Supplementary file 5 — Supporting Information 5 Table S1: STROBE‐MR checklist of recommended items to address in reports of Mendelian randomization studies. [file IJOG-2026-2921181-s009.docx]

**Table S1.** STROBE-MR checklist of recommended items to address in reports of Mendelian randomization studies.

| **Item No.** | **Section** | **Checklist item** | **Page No.** | **Relevant text from manuscript** |
| --- | --- | --- | --- | --- |
| 1 | **TITLE and ABSTRACT** | Indicate Mendelian randomization (MR) as the study’s design in the title and/or the abstract if that is a main purpose of the study | 1 | Causal effects of yogurt intake on gut microbiota: a European Mendelian randomization study |
|  | **INTRODUCTION** |  |  |  |
| 2 | **Background** | Explain the scientific background and rationale for the reported study. What is the exposure? Is a potential causal relationship between exposure and outcome plausible? Justify why MR is a helpful method to address the study question | 2 | For confounding exposures like lifestyle, MR could provide larger sample size and stronger evidence … There were few randomized studies and no MR studies between yogurt and gut microbiota. |
| 3 | **Objectives** | State specific objectives clearly, including pre-specified causal hypotheses (if any). State that MR is a method that, under specific assumptions, intends to estimate causal effects | 2 | We utilized UVMR and MVMR analyses to assess the causality between yogurt intake and gut microbiota |
|  | **METHODS** |  |  |  |
| 4 | **Study design and data sources** | Present key elements of the study design early in the article. Consider including a table listing sources of data for all phases of the study. For each data source contributing to the analysis, describe the following: |  |  |
|  | a) | Setting: Describe the study design and the underlying population, if possible. Describe the setting, locations, and relevant dates, including periods of recruitment, exposure, follow-up, and data collection, when available. | 2 | We accessed 3 datasets related to yogurt from MRC-IEU consortium, and 211 related to gut microbiota from MiBioGen consortium as Table 1. |
|  | b) | Participants: Give the eligibility criteria, and the sources and methods of selection of participants. Report the sample size, and whether any power or sample size calculations were carried out prior to the main analysis | 2 | The (yogurt) results contained 64949 individuals with European ancestry …We selected 14306 European individuals to calculate mbQTL. |
|  | c) | Describe measurement, quality control and selection of genetic variants | 3 | Balancing the sample size and statistical power, IVs were selected by following procedures. |
|  | d) | For each exposure, outcome, and other relevant variables, describe methods of assessment and diagnostic criteria for diseases | 2 | Yogurt intake contained how many pots (half, 1, 2, 3 or more) of yogurt (plain or flavored) were had per day by 97434 yogurt consumers |
|  | e) | Provide details of ethics committee approval and participant informed consent, if relevant | 6 | No specific ethical approval was required for this study since the summary-level GWAS data were publicly accessible. |
| 5 | **Assumptions** | Explicitly state the three core IV assumptions for the main analysis (relevance, independence and exclusion restriction) as well assumptions for any additional or sensitivity analysis | 3 | A variety of approaches were employed to evaluate the significance and sensitivity of the causality between yogurt intake and gut microbiota. |
| 6 | **Statistical methods: main analysis** | Describe statistical methods and statistics used |  |  |
|  | a) | Describe how quantitative variables were handled in the analyses (i.e., scale, units, model) | 2 | Yogurt intake contained how many pots of yogurt were had per day … mbQTL linked fecal microbiotic abundance to host genome-wide genotypes. |
|  | b) | Describe how genetic variants were handled in the analyses and, if applicable, how their weights were selected | 2 | MRC-IEU GWAS pipeline curated and outputted 3 exposure GWAS datasets |
|  | c) | Describe the MR estimator (e.g. two-stage least squares, Wald ratio) and related statistics. Detail the included covariates and, in case of two-sample MR, whether the same covariate set was used for adjustment in the two samples | 3 | *P* was calculated by MR Egger, weighted median, IVW, weighted mode and simple mode for multiple IVs …As for single IV results, we could only apply Wald ratio method. |
|  | d) | Explain how missing data were addressed | 2-3 | Except for 15 unknowns, 196 taxa of gut microbiota were identified. |
|  | e) | If applicable, indicate how multiple testing was addressed | 3 | Finally, all results were subjected to Benjamini-Hochberg (BH)[25] false discovery rate (FDR) correction, adopting an adjusted significance threshold of P < 0.05. |
| 7 | **Assessment of assumptions** | Describe any methods or prior knowledge used to assess the assumptions or justify their validity | 3 | For multiple IVs, it was determined by a specific method under different circumstances ... In MVMR, we utilized MV-IVW to estimate the direct effect. |
| 8 | **Sensitivity analyses and additional analyses** | Describe any sensitivity analyses or additional analyses performed (e.g. comparison of effect estimates from different approaches, independent replication, bias analytic techniques, validation of instruments, simulations) | 3 | Heterogeneity statistics, horizontal pleiotropy and Steiger directionality test were performed …F-statistic was calculated to test for weak instruments. |
| 9 | **Software and pre-registration** |  |  |  |
|  | a) | Name statistical software and package(s), including version and settings used | 6 | All analyses in this study were performed by R packages TwoSampleMR (version 0.5.7) and MVMR (version 0.4) in R (version 4.3.1) and RStudio software (version 6.0.421) . |
|  | b) | State whether the study protocol and details were pre-registered (as well as when and where) |  | N/A |
|  | **RESULTS** |  |  |  |
| 10 | **Descriptive data** |  |  |  |
|  | a) | Report the numbers of individuals at each stage of included studies and reasons for exclusion. Consider use of a flow diagram | 2 | The results contained 64949 individuals with European ancestry, including 15300 cases and 49649 controls in low-fat yogurt, and 3391 cases and 61558 controls in full-fat yogurt. |
|  | b) | Report summary statistics for phenotypic exposure(s), outcome(s), and other relevant variables (e.g. means, SDs, proportions) | 4 | There were 12 taxa of gut microbiota causally related to yogurt intake, as Table S5. |
|  | c) | If the data sources include meta-analyses of previous studies, provide the assessments of heterogeneity across these studies |  | N/A |
|  | d) | For two-sample MR:  i.  Provide justification of the similarity of the genetic variant-exposure associations between the exposure and outcome samples  ii.  Provide information on the number of individuals who overlap between the exposure and outcome studies | 4 | We obtained 13 SNPs for UVMR and 16 for MVMR following the IVs selecting procedures as Table S2 and S3. |
| 11 | **Main results** |  |  |  |
|  | a) | Report the associations between genetic variant and exposure, and between genetic variant and outcome, preferably on an interpretable scale | 4 | Then we summarized 1 class, 1 family and 4 specific genera by LPSN taxonomy, as shown in Fig 4 and Table 2. |
|  | b) | Report MR estimates of the relationship between exposure and outcome, and the measures of uncertainty from the MR analysis, on an interpretable scale, such as odds ratio or relative risk per SD difference | 4 | They were the genus *Haemophilus* (OR = 2.08, 95% CI: 1.27-3.41, *P* = 3.50 × 10^-3^) … |
|  | c) | If relevant, consider translating estimates of relative risk into absolute risk for a meaningful time period |  | N/A |
|  | d) | Consider plots to visualize results (e.g. forest plot, scatterplot of associations between genetic variants and outcome versus between genetic variants and exposure) | 15 | Fig 4. Forest plot of summarized results in UVMR and MVMR. OR and *P* were calculated by IVW method … |
| 12 | **Assessment of assumptions** |  |  |  |
|  | a) | Report the assessment of the validity of the assumptions | 4 | After eliminating biases of heterogeneity (Fig 2, S1 and Table S4), horizontal pleiotropy, weak instrument and causal direction (Table S4). |
|  | b) | Report any additional statistics (e.g., assessments of heterogeneity across genetic variants, such as *I^2^*, Q statistic or E-value) | 13 | Fig 2. Leave-one-out analysis of summarized microbiota in UVMR. Calculating without each SNP successively, the overall results were consistent and indicated no heterogeneity. |
| 13 | **Sensitivity analyses and additional analyses** |  |  |  |
|  | a) | Report any sensitivity analyses to assess the robustness of the main results to violations of the assumptions | 4 | After eliminating biases of heterogeneity (Fig 2, S1 and Table S4), horizontal pleiotropy, weak instrument and causal direction (Table S4). |
|  | b) | Report results from other sensitivity analyses or additional analyses | 14 | Fig 3. Scatter plot of summarized microbiota in UVMR. The tendencies were consistent in all 5 regression methods. |
|  | c) | Report any assessment of direction of causal relationship (e.g., bidirectional MR) | 4 | After eliminating biases of heterogeneity (Fig 2, S1 and Table S4), horizontal pleiotropy, weak instrument and causal direction (Table S4). |
|  | d) | When relevant, report and compare with estimates from non-MR analyses |  | N/A |
|  | e) | Consider additional plots to visualize results (e.g., leave-one-out analyses) | 13 | Fig 2. Leave-one-out analysis of summarized microbiota in UVMR. |
|  | **DISCUSSION** |  |  |  |
| 14 | **Key results** | Summarize key results with reference to study objectives | 6 | Yogurt intake causally increased the abundance of *Haemophilus*, *Clostridium sensu stricto_1* and *Peptostreptococcaceae*, and decreased ... |
| 15 | **Limitations** | Discuss limitations of the study, taking into account the validity of the IV assumptions, other sources of potential bias, and imprecision. Discuss both direction and magnitude of any potential bias and any efforts to address them | 4-5 | There were still several limitations in our study … |
| 16 | **Interpretation** |  |  |  |
|  | a) | Meaning: Give a cautious overall interpretation of results in the context of their limitations and in comparison with other studies | 5 | These alterations were similar to several studies about probiotic, prebiotic and synbiotic treatment … |
|  | b) | Mechanism: Discuss underlying biological mechanisms that could drive a potential causal relationship between the investigated exposure and the outcome, and whether the gene-environment equivalence assumption is reasonable. Use causal language carefully, clarifying that IV estimates may provide causal effects only under certain assumptions | 5 | They assumed the SCFAs and branched chain hydroxy acids as metabolic mediator to benefit energy metabolism. |
|  | c) | Clinical relevance: Discuss whether the results have clinical or public policy relevance, and to what extent they inform effect sizes of possible interventions | 5 | Yogurt intake increased the abundance of group B vitamins, peptides and minerals to strengthen the beneficial remodeling for intestinal environment |
| 17 | **Generalizability** | Discuss the generalizability of the study results (a) to other populations, (b) across other exposure periods/timings, and (c) across other levels of exposure | 5 | We couldn’t make conclusion in other ethnic populations since the European data source. |
|  | **OTHER INFORMATION** |  |  |  |
| 18 | **Funding** | Describe sources of funding and the role of funders in the present study and, if applicable, sources of funding for the databases and original study or studies on which the present study is based | 6 | This work was supported by Key Project of Scientific Research from Jiangsu Commission of Health [ZDB2020026] … |
| 19 | **Data and data sharing** | Provide the data used to perform all analyses or report where and how the data can be accessed, and reference these sources in the article. Provide the statistical code needed to reproduce the results in the article, or report whether the code is publicly accessible and if so, where | 6 | All GWAS datasets and software in this study are publicly available. The summary results of yogurt intake (GWAS ID: ukb-b-7753, UKB ID: 102090) … |
| 20 | **Conflicts of Interest** | All authors should declare all potential conflicts of interest | 6 | The authors declare no conflict of interest. |

This checklist is copyrighted by the Equator Network under the Creative Commons Attribution 3.0 Unported (CC BY 3.0) license.
