# Supplementary material for: Causal Effects of Yogurt Intake on Gut Microbiota: A European Mendelian Randomization Study
Source: Int J Genomics. 2026 Mar 3;2026:2921181. doi: 10.1155/ijog/2921181 (PMC12957542; doi:10.1155/ijog/2921181)
Supplement: Supplementary file 6 — Supporting Information 6 Table S2: SNP information in UVMR and MVMR, including chromosomal location, allele, and eaf. [file IJOG-2026-2921181-s006.docx]

**Table S2.** SNPs information in UVMR and MVMR, including chromosomal location, allele and eaf.

| exposure | snp | chromosome | effect allele | other allele | eaf |
| --- | --- | --- | --- | --- | --- |
| Yogurt intake | rs7157038 | 14:93202028 | C | T | 0.32 |
|  | rs392542 | 3:120567631 | A | G | 0.66 |
|  | rs3741434 | 12:53605344 | C | T | 0.14 |
|  | rs28614087 | 7:7887131 | A | C | 0.45 |
|  | rs2819017 | 1:244824192 | T | C | 0.10 |
|  | rs2344658 | 2:45764419 | A | C | 0.01 |
|  | rs150992808 | 1:47557624 | A | G | 0.04 |
|  | rs144143483 | 4:172083334 | C | A | 0.01 |
|  | rs11678849 | 2:5991393 | A | T | 0.31 |
|  | rs11521361 | 9:23249559 | A | T | 0.21 |
|  | rs113580100 | 12:11544624 | G | A | 0.04 |
|  | rs113524166 | 6:102633322 | T | G | 0.01 |
|  | rs10505667 | 8:137943581 | T | C | 0.44 |
| Multivariable low-fat & full-fat yogurt | rs10975896 | 9:6916272 | G | A | 0.62 |
|  | rs113459593 | 7:135844093 | T | C | 0.21 |
|  | rs11521361 | 9:23249561 | A | T | 0.21 |
|  | rs11525897 | 8:32863895 | C | G | 0.53 |
|  | rs1250593 | 10:79235799 | A | G | 0.69 |
|  | rs2363980 | 3:191233406 | C | T | 0.68 |
|  | rs2408654 | 5:56986719 | C | T | 0.21 |
|  | rs57966852 | 7:146058248 | A | G | 0.17 |
|  | rs5997275 | 22:27585409 | A | G | 0.20 |
|  | rs61871474 | 10:125783708 | G | A | 0.13 |
|  | rs72884403 | 18:13566910 | C | T | 0.39 |
|  | rs7612232 | 3:172650293 | G | A | 0.75 |
|  | rs76450551 | 1:21308484 | A | G | 0.20 |
|  | rs8012845 | 14:104674096 | G | A | 0.55 |
|  | rs9309222 | 2:51940625 | T | C | 0.30 |
|  | rs9869438 | 3:1132460 | C | G | 0.20 |
