# Supplementary material for: Causal Effects of Yogurt Intake on Gut Microbiota: A European Mendelian Randomization Study
Source: Int J Genomics. 2026 Mar 3;2026:2921181. doi: 10.1155/ijog/2921181 (PMC12957542; doi:10.1155/ijog/2921181)
Supplement: Supplementary file 7 — Supporting Information 7 Table S3: SNP statistics in UVMR an d MVMR, including β, se, p, and F statistic. [file IJOG-2026-2921181-s007.docx]

**Table S3.** SNPs statistics in UVMR and MVMR, including β, se, *P* and F statistic.

| exposure | snp | β | se | *P* | F |
| --- | --- | --- | --- | --- | --- |
| Yogurt intake | rs7157038 | 2.61E-02 | 5.69E-03 | 4.70E-06 | 19.25 |
|  | rs392542 | 2.74E-02 | 5.60E-03 | 9.90E-07 | 21.96 |
|  | rs3741434 | -3.55E-02 | 7.60E-03 | 3.00E-06 | 19.83 |
|  | rs28614087 | 2.45E-02 | 5.39E-03 | 5.60E-06 | 19.27 |
|  | rs2819017 | 4.17E-02 | 8.92E-03 | 2.90E-06 | 20.05 |
|  | rs2344658 | 2.06E-01 | 4.00E-02 | 2.70E-07 | 36.89 |
|  | rs150992808 | 6.68E-02 | 1.42E-02 | 2.50E-06 | 20.73 |
|  | rs149870452 | -9.70E-02 | 2.00E-02 | 1.30E-06 | 22.18 |
|  | rs144143483 | 1.15E-01 | 2.52E-02 | 4.60E-06 | 23.30 |
|  | rs11678849 | 2.77E-02 | 5.97E-03 | 3.50E-06 | 21.31 |
|  | rs11521361 | 3.27E-02 | 6.58E-03 | 6.70E-07 | 22.76 |
|  | rs113580100 | 6.32E-02 | 1.34E-02 | 2.40E-06 | 21.71 |
|  | rs113524166 | 1.27E-01 | 2.80E-02 | 5.70E-06 | 22.50 |
|  | rs10505667 | -2.43E-02 | 5.37E-03 | 5.80E-06 | 18.92 |
| Multivarite low-fat yogurt | rs10975896 | 1.10E-02 | 2.41E-03 | 5.40E-06 | N/A |
|  | rs113459593 | 1.29E-02 | 2.92E-03 | 9.80E-06 |  |
|  | rs11521361 | 1.33E-02 | 2.90E-03 | 4.90E-06 |  |
|  | rs11525897 | -1.07E-02 | 2.36E-03 | 6.10E-06 |  |
|  | rs1250593 | -1.25E-02 | 2.54E-03 | 9.10E-07 |  |
|  | rs2363980 | -1.13E-02 | 2.53E-03 | 7.60E-06 |  |
|  | rs2408654 | -4.96E-04 | 2.90E-03 | 8.60E-01 |  |
|  | rs2517506 | -1.54E-02 | 3.41E-03 | 5.80E-06 |  |
|  | rs317656 | 1.17E-02 | 2.61E-03 | 7.70E-06 |  |
|  | rs57966852 | -1.58E-02 | 3.16E-03 | 6.20E-07 |  |
|  | rs5997275 | 1.35E-02 | 2.95E-03 | 4.40E-06 |  |
|  | rs61871474 | 6.41E-04 | 3.50E-03 | 8.50E-01 |  |
|  | rs6888979 | 2.12E-03 | 2.58E-03 | 4.10E-01 |  |
|  | rs72884403 | -1.07E-02 | 2.41E-03 | 9.60E-06 |  |
|  | rs7612232 | -2.78E-03 | 2.78E-03 | 3.20E-01 |  |
|  | rs76450551 | -3.30E-03 | 2.96E-03 | 2.70E-01 |  |
|  | rs8012845 | 1.13E-02 | 2.39E-03 | 2.20E-06 |  |
|  | rs9309222 | 1.14E-02 | 2.57E-03 | 8.80E-06 |  |
|  | rs9604488 | -2.97E-03 | 2.90E-03 | 3.10E-01 |  |
|  | rs9869438 | 1.38E-02 | 2.95E-03 | 2.90E-06 |  |
| Multivarite full-fat yogurt | rs10975896 | 4.47E-04 | 1.27E-03 | 7.20E-01 |  |
|  | rs113459593 | 9.81E-04 | 1.54E-03 | 5.20E-01 |  |
|  | rs11521361 | 2.40E-03 | 1.53E-03 | 1.20E-01 |  |
|  | rs11525897 | 3.17E-03 | 1.24E-03 | 1.10E-02 |  |
|  | rs1250593 | 7.54E-04 | 1.34E-03 | 5.70E-01 |  |
|  | rs2363980 | -1.39E-03 | 1.33E-03 | 3.00E-01 |  |
|  | rs2408654 | 7.11E-03 | 1.53E-03 | 3.50E-06 |  |
|  | rs2517506 | 1.54E-03 | 1.80E-03 | 3.90E-01 |  |
|  | rs317656 | -1.99E-03 | 1.38E-03 | 1.50E-01 |  |
|  | rs57966852 | 2.60E-03 | 1.67E-03 | 1.20E-01 |  |
|  | rs5997275 | -4.63E-04 | 1.55E-03 | 7.70E-01 |  |
|  | rs61871474 | 9.10E-03 | 1.84E-03 | 8.10E-07 |  |
|  | rs6888979 | -6.26E-03 | 1.36E-03 | 3.90E-06 |  |
|  | rs72884403 | 1.66E-03 | 1.27E-03 | 1.90E-01 |  |
|  | rs7612232 | 6.74E-03 | 1.46E-03 | 4.10E-06 |  |
|  | rs76450551 | 6.95E-03 | 1.56E-03 | 8.40E-06 |  |
|  | rs8012845 | -1.54E-04 | 1.26E-03 | 9.00E-01 |  |
|  | rs9309222 | -3.14E-03 | 1.35E-03 | 2.00E-02 |  |
|  | rs9604488 | 7.22E-03 | 1.53E-03 | 2.20E-06 |  |
|  | rs9869438 | 1.59E-04 | 1.56E-03 | 9.20E-01 |  |
