# Supplementary material for: Causal Effects of Yogurt Intake on Gut Microbiota: A European Mendelian Randomization Study
Source: Int J Genomics. 2026 Mar 3;2026:2921181. doi: 10.1155/ijog/2921181 (PMC12957542; doi:10.1155/ijog/2921181)
Supplement: Supplementary file 8 — Supporting Information 8 Table S4: Heterogeneity statistics (IVW and MR Egger), horizontal pleiotropy test, F statistic, Steiger directionality test, and p adjusted (BH) of UVMR and MVMR. [file IJOG-2026-2921181-s008.docx]

**Table S4.** Heterogeneity statistics (IVW and MR Egger), horizontal pleiotropy test, F statistic, Steiger directionality test and *P* adjusted (BH) of UVMR and MVMR.

| exposure | outcome | nsnp | heterogeneity pval | | pleiotropy pval | F | Steiger pval | padj |
| --- | --- | --- | --- | --- | --- | --- | --- | --- |
|  |  |  | IVW | MR Egger |  |  |  |  |
| Yogurt intake | class *Betaproteobacteria* | 7 | 0.78 | 0.78 | 0.43 | N/A | 4.75E-04 | 0.75 |
|  | class *Deltaproteobacteria* | 7 | 0.47 | 0.36 | 0.84 |  | 6.16E-03 | 0.09 |
|  | family *Clostridiaceae1* | 7 | 0.86 | 0.79 | 0.66 |  | 2.30E-03 | 0.12 |
|  | family *Desulfovibrionaceae* | 7 | 0.48 | 0.36 | 0.91 |  | 5.01E-03 | 0.09 |
|  | family *Pasteurellaceae* | 6 | 0.90 | 0.81 | 0.98 |  | 5.93E-03 | 0.30 |
|  | family *Peptostreptococcaceae* | 7 | 0.82 | 0.72 | 0.98 |  | 2.68E-04 | 0.40 |
|  | genus *Bilophila* | 7 | 0.67 | 0.57 | 0.70 |  | 4.79E-03 | 0.22 |
|  | genus *Clostridium sensu stricto_1* | 7 | 0.84 | 0.76 | 0.73 |  | 3.81E-03 | 0.09 |
|  | genus *Haemophilus* | 6 | 0.92 | 0.86 | 0.77 |  | 9.59E-03 | 0.12 |
|  | genus *Ruminococcaceae UCG-011* | 6 | 0.75 | 0.62 | 1.00 |  | 3.04E-03 | 0.40 |
|  | order *Desulfovibrionales* | 7 | 0.46 | 0.34 | 0.89 |  | 5.62E-03 | 0.09 |
|  | order *Pasteurellales* | 6 | 0.90 | 0.81 | 0.98 |  | 5.93E-03 | 0.30 |
| Low-fat yogurt | class *Methanobacteria* | 16 | 0.92 | N/A | | 13.48 | 4.45E-06 | 0.93 |
|  | family *Methanobacteriaceae* |  | 0.92 |  |  |  | 4.45E-06 | 0.93 |
|  | genus *Eubacterium ruminantium group* |  | 1.00 |  |  |  | 7.16E-06 | 0.10 |
|  | order *Methanobacteriales* |  | 0.92 |  |  |  | 4.45E-06 | 0.93 |
|  | phylum *Euryarchaeota* |  | 0.89 |  |  |  | 1.04E-05 | 0.93 |
