# Supplementary material for: Causal Effects of Yogurt Intake on Gut Microbiota: A European Mendelian Randomization Study
Source: Int J Genomics. 2026 Mar 3;2026:2921181. doi: 10.1155/ijog/2921181 (PMC12957542; doi:10.1155/ijog/2921181)
Supplement: Supplementary file 9 — Supporting Information 9 Table S5: All causal results of UVMR and MVMR, including β, se, and p calculated by five methods. [file IJOG-2026-2921181-s003.docx]

**Table S5.** All causal results of UVMR and MVMR, including β, se and *P* calculated by 5 methods.

| exposure | outcome | nsnp | method | β | se | *P* |
| --- | --- | --- | --- | --- | --- | --- |
| Yogurt intake | class *Betaproteobacteria* | 7 | MR Egger | -1.01 | 0.78 | 0.252 |
|  |  |  | Weighted median | -0.28 | 0.22 | 0.210 |
|  |  |  | IVW | -0.35 | 0.18 | 0.046 |
|  |  |  | Simple mode | -0.26 | 0.32 | 0.446 |
|  |  |  | Weighted mode | -0.26 | 0.30 | 0.428 |
|  | class *Deltaproteobacteria* | 7 | MR Egger | -0.41 | 0.87 | 0.652 |
|  |  |  | Weighted median | -0.32 | 0.26 | 0.220 |
|  |  |  | IVW | -0.60 | 0.19 | 0.001 |
|  |  |  | Simple mode | -0.22 | 0.43 | 0.635 |
|  |  |  | Weighted mode | -0.19 | 0.39 | 0.642 |
|  | family *Clostridiaceae1* | 7 | MR Egger | 0.20 | 0.83 | 0.823 |
|  |  |  | Weighted median | 0.56 | 0.25 | 0.025 |
|  |  |  | IVW | 0.57 | 0.19 | 0.003 |
|  |  |  | Simple mode | 0.67 | 0.37 | 0.121 |
|  |  |  | Weighted mode | 0.65 | 0.37 | 0.126 |
|  | family *Desulfovibrionaceae* | 7 | MR Egger | -0.49 | 0.87 | 0.598 |
|  |  |  | Weighted median | -0.33 | 0.27 | 0.228 |
|  |  |  | IVW | -0.59 | 0.19 | 0.002 |
|  |  |  | Simple mode | -0.23 | 0.40 | 0.592 |
|  |  |  | Weighted mode | -0.20 | 0.43 | 0.661 |
|  | family *Pasteurellaceae* | 6 | MR Egger | 0.66 | 1.38 | 0.659 |
|  |  |  | Weighted median | 0.56 | 0.31 | 0.073 |
|  |  |  | IVW | 0.62 | 0.25 | 0.013 |
|  |  |  | Simple mode | 0.36 | 0.42 | 0.433 |
|  |  |  | Weighted mode | 0.36 | 0.44 | 0.449 |
|  | family *Peptostreptococcaceae* | 7 | MR Egger | 0.44 | 0.79 | 0.601 |
|  |  |  | Weighted median | 0.50 | 0.23 | 0.033 |
|  |  |  | IVW | 0.42 | 0.18 | 0.020 |
|  |  |  | Simple mode | 0.52 | 0.38 | 0.214 |
|  |  |  | Weighted mode | 0.52 | 0.36 | 0.203 |
|  | genus *Bilophila* | 7 | MR Egger | -0.18 | 0.91 | 0.848 |
|  |  |  | Weighted median | -0.42 | 0.26 | 0.111 |
|  |  |  | IVW | -0.55 | 0.20 | 0.007 |
|  |  |  | Simple mode | -0.26 | 0.43 | 0.571 |
|  |  |  | Weighted mode | -0.30 | 0.42 | 0.503 |
|  | genus *Clostridium sensu stricto_1* | 7 | MR Egger | 0.32 | 0.84 | 0.721 |
|  |  |  | Weighted median | 0.69 | 0.26 | 0.008 |
|  |  |  | IVW | 0.61 | 0.19 | 0.002 |
|  |  |  | Simple mode | 0.72 | 0.39 | 0.114 |
|  |  |  | Weighted mode | 0.72 | 0.36 | 0.089 |
|  | genus *Haemophilus* | 6 | MR Egger | 1.17 | 1.40 | 0.452 |
|  |  |  | Weighted median | 0.68 | 0.31 | 0.028 |
|  |  |  | IVW | 0.73 | 0.25 | 0.004 |
|  |  |  | Simple mode | 0.58 | 0.45 | 0.260 |
|  |  |  | Weighted mode | 0.58 | 0.46 | 0.266 |
|  | genus *Ruminococcaceae UCG-011* | 6 | MR Egger | -0.90 | 2.19 | 0.704 |
|  |  |  | Weighted median | -1.19 | 0.49 | 0.015 |
|  |  |  | IVW | -0.91 | 0.39 | 0.021 |
|  |  |  | Simple mode | -1.33 | 0.73 | 0.129 |
|  |  |  | Weighted mode | -1.32 | 0.75 | 0.139 |
|  | order *Desulfovibrionales* | 7 | MR Egger | -0.48 | 0.88 | 0.611 |
|  |  |  | Weighted median | -0.32 | 0.26 | 0.209 |
|  |  |  | IVW | -0.60 | 0.19 | 0.001 |
|  |  |  | Simple mode | -0.22 | 0.44 | 0.634 |
|  |  |  | Weighted mode | -0.20 | 0.40 | 0.634 |
|  | order *Pasteurellales* | 7 | MR Egger | 0.66 | 1.38 | 0.659 |
|  |  |  | Weighted median | 0.56 | 0.31 | 0.071 |
|  |  |  | IVW | 0.62 | 0.25 | 0.013 |
|  |  |  | Simple mode | 0.36 | 0.43 | 0.443 |
|  |  |  | Weighted mode | 0.36 | 0.46 | 0.465 |
| Low-fat yogurt | class *Methanobacteria* | 16 | MV-IVW | 1.12 | 0.48 | 0.041 |
|  | family *Methanobacteriaceae* |  |  | 1.12 | 0.48 | 0.041 |
|  | genus *Eubacterium ruminantium group* |  |  | 0.91 | 0.19 | 4.76E-04 |
|  | order *Methanobacteriales* |  |  | 1.12 | 0.48 | 0.041 |
|  | phylum *Euryarchaeota* |  |  | 1.15 | 0.50 | 0.043 |
